# Artificial intelligence adoption and corporate ESG performance: evidence from a refined large language model

**DOI:** 10.3389/frai.2025.1691468

**Published:** 2025-10-27

**Authors:** Lihao Shen, Zhengrong Li, Yongqing Liang, Yiqiang Feng, Zhanyu Zhang

**Affiliations:** ^1^College of Social Sciences, University of California, Los Angeles, Los Angeles, CA, United States; ^2^School of Economics, Central University of Finance and Economics, Beijing, China; ^3^College of Design and Engineering, National University of Singapore, Singapore, Singapore; ^4^China Center for Internet Economy Research, Central University of Finance and Economics, Beijing, China

**Keywords:** artificial intelligence, ESG performance, Large Language Models (LLMs), corporate sustainability, technological integration

## Abstract

**Introduction:**

The convergence of artificial intelligence (AI) and Environmental, Social, and Governance (ESG) objectives has attracted growing academic and policy interest but remains empirically underexplored due to challenges in accurately measuring firm-level AI adoption.

**Methods:**

This study refines the LLM-based framework by employing a domain-adapted model (Qwen2.5-72B) and a granular classification scheme to distinguish genuine “Applied” AI technologies from rhetorical mentions in corporate disclosures. Using data from Chinese A-share listed firms between 2009 and 2022, we construct a credible indicator of AI adoption and examine its impact on ESG performance.

**Results and discussion:**

The results reveal a robust positive relationship between AI adoption and ESG outcomes, primarily driven by enhanced green innovation and improved internal control quality. These effects are more pronounced among large and technology-intensive firms. Consistent with the Resource-Based View and the Technology–Organization–Environment framework, our findings underscore the importance of complementary assets and absorptive capacity in realizing the sustainability potential of AI. This study provides credible evidence on how and for whom AI fosters corporate sustainability, introduces a transparent approach to measuring authentic technology adoption, and highlights the emerging “digital ESG divide” with implications for targeted policy interventions.

## 1 Introduction

As environmental challenges intensify and resource constraints tighten, accelerating the green transformation of development models has become imperative for achieving ecological protection, promoting the efficient use of resources, and advancing broader societal progress toward sustainable development goals. The greening of economic activity is emerging as a defining trend of the ongoing scientific and technological revolution and the accompanying wave of industrial transformation, with heightened demands for social responsibility reshaping corporate strategies and business philosophies. In this new stage of development, firms must overcome resource and environmental constraints, transform production paradigms, and embed the principles of ecological civilization across all facets of production, operations, and governance. Environmental, Social, and Governance (ESG) considerations—capturing a firm’s performance in these three dimensions—have thus become a critical benchmark for assessing corporate commitment to green and sustainable development. In China, the Securities Regulatory Commission has introduced a series of policies and regulations aimed at strengthening corporate ESG responsibilities. Against this backdrop of rising scrutiny from both investors and policymakers, enhancing ESG performance has become an unavoidable strategic imperative for contemporary enterprises.

In recent years, artificial intelligence (AI) has not only enhanced firms’ production and managerial efficiency (Chowdhury et al., 2022; Babina et al., 2024), but has also exhibited considerable potential and technological advantages in addressing ESG-related challenges (Chen et al., 2024). For instance, AI facilitates the processing of vast and complex ESG-related datasets, thereby improving both the quality and the credibility of ESG disclosures, while also enabling continuous monitoring of ESG practices to support real-time risk management and the identification of new opportunities. Moreover, by leveraging real-time data collection and intelligent analytics, AI can issue early warnings regarding energy consumption and pollutant emissions, thereby mitigating environmental degradation. At the same time, AI fosters technological innovation, strengthens firms’ capacity to adapt to external environmental pressures, and supports the pursuit of long-term sustainable operations. Although practical applications underscore AI’s promise in advancing corporate ESG objectives, empirical evidence remains relatively scarce, leaving the mechanisms and extent of AI’s influence on ESG performance insufficiently understood. This gap highlights the need for focused research on this emerging technology and its implications for corporate ESG responsibilities.

Firms are currently under dual pressures to adopt new technologies and to improve sustainability disclosure and performance. In China, this institutional pressure has evolved dynamically over two decades. The process began with early exchange-level guidance that encouraged voluntary reporting, such as the Shenzhen Stock Exchange’s CSR instructions (Shenzhen Stock Exchange, 2006) and the Shanghai Stock Exchange’s environmental disclosure guidance (Shanghai Stock Exchange, 2008). This was followed by a gradual shift toward more binding requirements, particularly for heavily polluting industries after 2016. This long-term evolution culminated in the comprehensive, scope-based mandatory sustainability reporting rules introduced by all three major exchanges in 2024 (Beijing Stock Exchange, 2024; Shanghai Stock Exchange, 2024; Shenzhen Stock Exchange, 2024). Against this backdrop, and amid competitive pressure to deploy transformative technologies such as artificial intelligence (AI)—systems capable of learning from data to make predictions, recommendations, or decisions—(Brynjolfsson and McAfee, 2014; Acemoglu and Restrepo, 2018), AI has moved from experimentation to operations, supporting real-time monitoring, process optimization, and audit-ready records. This leads to our central question: does firm-level AI adoption translate into measurable improvements in Environmental, Social, and Governance (ESG) performance, and through which organizational channels? Credible answers require measuring authentic adoption—implemented use rather than generic narratives—and tracing how AI reshapes green innovation and internal controls that rating agencies record.

We define AI adoption as implemented, operational use embedded in workflows (e.g., monitoring, planning, anomaly detection), not merely aspirational statements in reports. Text-based indicators enable panel coverage but face a discourse–action gap when firms discuss AI without deploying it. Recent work therefore moves beyond keyword tallies toward Natural Language Processing (NLP) and Large Language Models (LLMs) for classification of disclosures, alongside official survey benchmarks of firm adoption (Di Vaio et al., 2020; US Census Bureau, 2024; Ante and Saggu, 2025). Building on this shift, we construct a refined, disclosure-based indicator targeting authentic (applied) adoption and align it with contemporaneous adoption evidence, allowing more credible tests of the AI–ESG link in a large panel of Chinese A-share firms (2009–2022).

As an innovative strategic resource, artificial intelligence not only holds substantial intrinsic value but also enables firms to develop distinctive resource portfolios by optimizing operational management and fostering novel production methods. Firms can achieve meaningful differentiation in increasingly competitive markets only by deploying such resources judiciously and transforming them into critical capabilities. Therefore, this study is grounded in strategic management and the economics of innovation, drawing on the Resource-Based View (RBV) and the Technology–Organization–Environment (TOE) framework (Tornatzky et al., 1990; Barney, 1991). These perspectives highlight that firms generate value by effectively mobilizing specific resources to develop and deploy capabilities. This integrative framework offers a robust theoretical foundation for unpacking the mechanisms through which the adoption of AI technologies influences firms’ ESG responsibilities. We then test two organizational channels—green innovation and internal control quality—and examine heterogeneity by firm size and technological intensity.

Despite growing interest, findings on the AI–ESG relationship remain unsettled because common proxies conflate implementation with intent. We address this measurement challenge with a refined LLM-based indicator of authentic adoption, detailed and validated in “Section 3,” and use it to test the relationship and its channels.

This paper contributes in three ways. First, we provide large-sample evidence that AI adoption improves firms’ ESG performance, and we show when and for whom the effect is stronger (larger and more technology-intensive firms). Second, we introduce a transparent, replicable text measure of authentic AI adoption that improves construct validity relative to keyword or patent proxies and is externally benchmarked. Third, we articulate and test two mechanisms—green innovation and internal control quality—explicitly tied to RBV and TOE, offering implications for managers aligning digital transformation with sustainability and for regulators designing disclosure/assurance regimes.

The remainder of the paper proceeds as follows: Section 2 reviews related literature and develops hypotheses; Section 3 details data and empirical strategy, including the refined indicator; Section 4 reports results and mechanism tests with robustness; Section 5 concludes.

## 2 Literature review and hypothesis development

### 2.1 Conceptualizing AI and its adoption challenges

Before assessing its impact, it is essential to first conceptualize AI itself. Adopting the updated OECD definition, we define an AI system as a machine-based system that, for explicit or implicit objectives, infers from received inputs how to generate outputs—such as predictions, content, recommendations, or decisions—that influence physical or virtual environments (OECD, 2024). Unlike traditional deterministic software, AI systems are designed to operate with varying degrees of autonomy and to improve their performance through experience (Russell and Norvig, 2022). This capacity for learning and adaptation—typically driven by machine learning algorithms—constitutes the defining hallmark of AI (Jordan and Mitchell, 2015).

Artificial intelligence serves as a key driver of technological innovation across diverse domains, including new product development, production process optimization, organizational transformation, and business model innovation. Compared with traditional technological advances, AI exhibits markedly more non-rivalrous and intangible characteristics, resulting in relatively low marginal production costs for AI-related goods and services (Yu et al., 2021). A substantial body of research has examined the economic, social, and environmental implications of AI adoption. With respect to its economic effects—particularly on growth and labor market dynamics—scholars generally concur that AI fosters macroeconomic expansion primarily by enhancing labor productivity across sectors and facilitating structural upgrades in the real economy. The pivotal mechanism underlying productivity gains lies in AI’s transformative impact on workforce skill composition (Acemoglu and Restrepo, 2020). In addition, a growing literature investigates the sustainability implications of AI technologies. Vinuesa et al. (2020) demonstrate that AI contributes to the attainment of multiple Sustainable Development Goals (SDGs), with particularly strong effects on environmental and social objectives. For instance, AI improves energy structure and enhances the efficiency of renewable energy utilization (Yin and Zeng, 2023) while supporting air quality monitoring and pollution source identification (Kaginalkar et al., 2021), thereby effectively curbing carbon emissions and mitigating environmental pollution intensity (Chen et al., 2024).

The unique characteristics of AI also present significant adoption challenges and risks for firms. The development and deployment of AI systems require substantial upfront investment in computational infrastructure, data storage, and, most critically, specialized human capital. Furthermore, AI’s reliance on vast datasets raises significant concerns related to data privacy, security, and potential algorithmic bias (Borenstein and Howard, 2021). These operational, financial, and ethical risks are not trivial; they require a robust governance and risk management framework, a topic of growing importance for both standard-setting bodies and regulators (International Organization for Standardization and International Electrotechnical Commission, 2023; National Institute of Standards and Technology (US) and U.S. Department of Commerce, 2024).

Moreover, owing to the absence of robust firm-level metrics for measuring AI adoption, much of the existing research has examined the micro-level sustainability implications of AI only indirectly—most often through proxies such as digital technologies, industrial robotics, or broader digital transformation initiatives (Fang et al., 2023). The literature most closely related to this study investigates the effects of digital transformation on corporate ESG performance (Fang et al., 2023), offering preliminary insights into the linkages between digital technologies and ESG outcomes. Yet, digital transformation captures the aggregate influence of a wide array of digital tools and applications. Without a more granular examination of specific technologies—particularly AI—it remains challenging to provide firms with clear guidance on harnessing digital tools for ESG advancement, let alone to unlock the full potential of AI in this domain.

Given these complexities, measuring AI adoption at the firm level is a non-trivial empirical challenge. We define AI “adoption” as the integration of AI tools into core organizational routines—such as monitoring, planning, detection, and optimization—so that it is the workflows themselves, rather than mere narratives, that generate observable outcomes (Melville et al., 2004; Comin and Hobijn, 2010; Davenport, 2018). Yet corporate disclosures often exaggerate the extent of implementation: aspirational language and boilerplate statements inflate simple mention-based metrics, introducing bias by conflating intent with actual use. This challenge reflects a well-documented phenomenon in legitimacy-seeking corporate communication (Hooghiemstra, 2000; Loughran and McDonald, 2011; Gentzkow et al., 2019) akin to the “greenwashing” risk widely discussed in the context of sustainable finance (Schwendner and Posth, 2024). Acknowledging this discourse—action gap, recent studies have moved beyond keyword counts and patent data, favoring NLP- and LLM-based classification of disclosures as well as official survey data that better capture actual adoption (Di Vaio et al., 2020; Jin et al., 2024; US Census Bureau, 2024; Ante and Saggu, 2025). Building on this approach, we construct a disclosure-based indicator refined to better isolate authentic AI adoption.

### 2.2 Theoretical framework and main hypotheses

This study aims to explore the impact mechanism of artificial intelligence (AI) adoption on ESG performance and how this impact varies across different corporate characteristics. To establish the theoretical foundation for this study, this paper will integrate the RBV, which focuses on internal resources and capabilities, with the TOE framework, which analyzes technology adoption scenarios from multiple dimensions.

#### 2.2.1 The resource-based view (RBV)

The RBV posits that a firm’s sustainable competitive advantage arises from the possession and effective deployment of strategic resources that are valuable, rare, inimitable, and non-substitutable (Barney, 1991). Such resources encompass not only tangible assets but, more critically, intangible assets—including technological expertise, organizational culture, and brand reputation—as well as the organizational processes that integrate and leverage these assets, that is, the firm’s capabilities.

A rich stream of literature has applied the RBV to IT adoption, arguing that intangible assets like a data-driven culture and specialized analytical skills are more critical than physical hardware (Mikalef and Gupta, 2021). We extend this logic to AI, a technology uniquely reliant on such intangible capabilities, which predicts larger returns where firms hold complementary assets—data, integration capability, and human capital—that enable learning from and scaling AI (Barney, 1991). Within this framework, AI should be understood not merely as a technological tool but as a highly valuable strategic resource capable of reshaping firms’ core capabilities.

#### 2.2.2 The technology-organization-environment (TOE) framework

Similarly, the TOE framework has a long tradition in explaining technology diffusion. It posits that adoption and its outcomes are a function of the technological context, organizational readiness, and the external environment (Tornatzky et al., 1990). We apply this lens to emphasize the role of absorptive capacity as a key moderator of AI’s impact (Cohen and Levinthal, 1990). It posits that a firm’s adoption decisions and their subsequent outcomes are shaped by three interrelated contextual factors. The technological context comprises characteristics of the focal technology, including its performance, complexity, compatibility, and cost. The organizational context encompasses firm-level attributes such as size, scope of operations, resource endowments, employee skill sets, and organizational structure. The environmental context captures external conditions in which the firm operates, including industry structure, competitive pressures, government regulation, and ESG-related expectations articulated by consumers and investors.

The TOE framework provides a useful lens for understanding the heterogeneity in AI adoption outcomes—that is, why firms implementing the same AI technology can achieve markedly different results. Among these factors, the organizational context is particularly important, offering a key theoretical explanation for the empirical patterns observed in this study. Specifically, it suggests that realizing the potential of AI to enhance ESG performance requires a corresponding organizational foundation capable of supporting and leveraging the technology effectively.

#### 2.2.3 AI adoption and ESG performance

A growing body of empirical work links AI adoption to higher ESG scores and greater green-innovation efficiency (Hussain et al., 2024; Liu et al., 2024; Wang et al., 2024; Xie et al., 2025). This aligns with a broader literature in corporate finance arguing that strong ESG performance can build stakeholder trust and social capital, which proves particularly valuable during times of crisis (Lins et al., 2017; Gillan et al., 2021). Yet, beyond the primary measurement challenge, two further frictions complicate inference. First, the returns to AI appear highly heterogeneous, concentrating where complementary assets and data infrastructures are already in place (Bresnahan et al., 2002). Second, endogeneity concerns loom large, as digital maturity may jointly drive both AI adoption and ESG performance.

We treat ESG performance as an organizational outcome. While disagreement across raters injects noise (Berg et al., 2022), the underlying construct reflects tangible actions that AI can influence. Given the promising evidence, deploying our more precise measure of authentic AI adoption should reveal a clearer, positive association.

*H1 (Main Effect)*: Firms with higher authentic AI adoption exhibit higher ESG performance.

### 2.3 Mechanism pathways and hypotheses

To unpack the main effect hypothesized above, we theorize two primary organizational pathways through which AI’s capabilities translate into observable ESG outcomes. We focus on the Environmental (E) and Governance (G) dimensions as they represent the most direct and empirically tractable channels. We argue that a firm’s performance in these pillars is driven by specific, measurable corporate actions: its capacity for green innovation and the quality of its internal control infrastructure. As a comprehensive review by Gillan et al. (2021) highlights, a firm’s environmental impact and its governance quality are central to modern analyses of corporate social responsibility. Therefore, we test these two pathways as analytically distinct channels.

#### 2.3.1 The green innovation channel

In research and development (R&D), AI accelerates the creation of environmentally friendly technologies and products by simulating novel materials, optimizing energy efficiency throughout supply chains, and mining vast bodies of environmental science literature and patent databases. These applications enhance a firm’s dynamic capabilities to address environmental challenges, thereby advancing the “Environmental” pillar of ESG. Rather than merely accelerating existing R&D, AI enables entirely new approaches to complex environmental problems. This aligns with the Porter Hypothesis, which posits that well-designed environmental pressures can trigger innovation that enhances competitiveness (Porter and van der Linde, 1995). For instance, firms can leverage AI in climate modeling to better forecast physical risks (Amnuaylojaroen, 2025), or use it to optimize supply chain logistics for reduced carbon emissions (Samuels, 2025). More directly, firms can use digital twins to simulate the lifecycle environmental impact of new products before prototyping, or employ machine learning models to accelerate the discovery of novel, sustainable materials (Vinuesa et al., 2020). These techniques systematically lower the cost and uncertainty of experimentation, thereby increasing the productivity of environmentally oriented R&D (Mikalef and Gupta, 2021; Kar et al., 2022). Recent studies confirm that stronger AI adoption is associated with higher green-innovation efficiency (Hussain et al., 2024; Wang et al., 2024; Xie et al., 2025).

*H2 (Green Innovation Channel)*: AI adoption increases a firm’s green innovation output, which in turn mediates the positive relationship between AI adoption and ESG performance.

#### 2.3.2 Internal control quality

A robust internal control system constitutes a cornerstone of the governance pillar of ESG. Extensive literature underscores its critical role in ensuring high-quality corporate governance, with seminal studies such as Doyle et al. (2007) empirically demonstrating that weaknesses in internal controls are linked to poor financial reporting quality—a direct manifestation of governance failure. AI has the potential to substantially strengthen this foundational system, moving beyond the static, rule-based alerts typical of traditional platforms to establish a more resilient and trustworthy governance framework.

This enhancement is operational rather than abstract, embedded within the firm’s core technological systems, primarily Enterprise Resource Planning (ERP) and Manufacturing Execution Systems (MES). While these systems effectively codify business processes, their native controls are generally limited to identifying known violations of pre-defined rules. AI adds a layer of intelligence, leveraging machine learning to analyze vast, cross-functional datasets and detect novel, complex, or collusive patterns of anomalous behavior that would otherwise remain undetected (Cui et al., 2022).

This transition from reactive to proactive monitoring fosters a more dynamic form of corporate governance, consistent with principles of “Trustworthy AI,” which emphasize reliability and accountability. Implementing AI-driven controls represents not merely a technical upgrade but a strategic investment in resilient compliance and cybersecurity infrastructure (Radanliev et al., 2025). These improvements operate within established enterprise architecture frameworks, such as The Open Group Architecture Framework (TOGAF), which structure governance and data management practices (The Open Group, 2018). The tangible outcomes—enhanced documentation quality, reduced compliance errors, and faster remediation—directly align with the metrics used by governance raters (Yang et al., 2020; Di Vaio et al., 2024; Li S. et al., 2024).

Modern assurance practices increasingly employ AI to validate evidence and mitigate information risk in sustainability reporting (Li N. et al., 2024). By enabling real-time monitoring and analysis of extensive internal operational data, AI allows firms to detect fraud, inefficiencies, and anomalous behaviors linked to environmental and social risks. In doing so, AI strengthens internal control and governance structures, thereby enhancing the “Governance” dimension of ESG and supporting the development of robust, transparent monitoring and reporting systems that are difficult for competitors to replicate.

*H3 (Internal Control Channel)*: AI adoption enhances the quality of a firm’s internal controls, which in turn mediates the positive relationship between AI adoption and ESG performance.

## 3 Empirical methodology and data

### 3.1 Model specification

To empirically test our hypotheses, we specify the following panel fixed-effects model, which allows us to control for all time-invariant firm-specific heterogeneity:


$$\mathrm{ES}G_{\mathrm{it}}=\alpha +\beta_{1}AI_{\mathrm{it}}+\beta_{k}X\prime_{\mathrm{it}}+\mu_{i}+\tau_{t}+\varepsilon_{\mathrm{it}}$$

In this specification, the subscript *i* denotes the firm and *t* denotes the year. The dependent variable, $\mathrm{ES}G_{\mathrm{it}}$ , is the corporate ESG performance score. $AI_{\mathrm{it}}$ is the dichotomous indicator for authentic AI adoption. The model includes a vector of firm-level control variables, denoted by $X{'}_{\mathrm{it}}$ , selected based on the corporate finance literature. We incorporate firm fixed effects ( $\mu_{i})$ to control for unobserved time-invariant characteristics and year fixed effects $\left(\tau_{t}\right)$ to account for shocks common to all firms. Standard errors are clustered at the firm level to account for serial correlation in the error term, $\varepsilon_{\mathrm{it}}$ .

### 3.2 Data and sample construction

Our study combines data from several standard sources for Chinese firms. We obtain firm-level financial and governance data from the China Stock Market & Accounting Research (CSMAR) database (Shenzhen CSMARData Technology Co., Ltd., 2024). The full texts of corporate annual reports, used to construct our AI adoption measure, are sourced from the CNINFO database (Shenzhen Securities Information Co., Ltd., 2024). Our primary measure of corporate ESG performance, the Huazheng ESG rating, is accessed through the Wind database (Sino-Securities Index Information Service (Shanghai) Co., Ltd., 2024).

Our sample period runs from 2009 to 2022. The starting year allows us to capture the nascent stages of AI discourse in a post-financial crisis environment, and 2022 is the most recent year for which complete data were available at the time of collection. We construct the final sample by applying several standard filters: we exclude firms in the financial industry, those designated as “ST” for financial distress, and any firm-year observations with missing data for our key variables. Finally, all continuous variables are winsorized at the 1st and 99th percentiles to mitigate the influence of outliers. These procedures yield a final unbalanced panel of 22,931 firm-year observations.

### 3.3 Variables and measurement

#### 3.3.1 Dependent variable: ESG performance

Our dependent variable is corporate ESG performance, measured using the Huazheng ESG rating index. We select this measure for several reasons. It is a comprehensive and widely utilized rating system for Chinese listed firms, covering a long time series consistent with our sample period. Its adoption in numerous recent high-quality academic studies establishes it as a credible and recognized benchmark for research on ESG in China (Shen et al., 2023). The system assigns a final grade on a nine-tier scale (from C to AAA), which we convert to a numerical score from 1 to 9, where higher values indicate superior ESG performance.

#### 3.3.2 Key explanatory variable: AI adoption

The construction of a credible measure of AI adoption is the core methodological contribution of this paper. Our process, which builds upon the foundational LLM-based framework of Jin et al. (2024), is designed specifically to address the “discourse-action gap” by integrating several key refinements to enhance precision and authenticity. The transparent, multi-step process is as follows.

First, for text corpus compilation, we source the annual reports of all Chinese A-share firms from 2009 to 2022 from CNINFO. We then extract text from the Management Discussion and Analysis (MD&A) section, as its forward-looking and strategic nature offers the highest signal-to-noise ratio for identifying genuine corporate strategy, as opposed to boilerplate sections.

Second, for model preparation, our process leverages a powerful, contemporary LLM, Qwen2.5-72B^1^, selected for its state-of-the-art performance on Chinese text. To optimize its capabilities for our specific domain, we conduct domain-adaptive pre-training. This involves continuing to train the base model on a large corpus of annual reports from a pre-sample period (2007–2008) to prevent data leakage, thereby equipping the model with a deep understanding of business-specific jargon and reporting context.

Third, the crucial innovation of our approach lies in the supervised fine-tuning with granular labels. This step moves decisively beyond a simple binary classification (AI vs. not AI). Our research team manually annotated over 38,000 sentences, assigning a label for the application status of the technology. We define three mutually exclusive statuses: “Applied” (for sentences describing technology already implemented or in current use), “Planned” (for sentences detailing future intentions or unrealized projects), and “General Mention” (for sentences discussing industry trends or definitions without reference to the firm’s own actions). This multi-dimensional labeling is essential for surgically isolating authentic adoption from corporate rhetoric.

Fourth, we conduct a rigorous performance validation to formally confirm our model’s enhanced precision. For this, we curated a challenging subsample of 500 ambiguous sentences where distinguishing true application from rhetoric is most difficult. Using human-adjudicated classifications as the ground truth, our fine-tuned model demonstrated a significantly higher precision rate in correctly identifying “Applied” sentences compared to baseline models. This result confirms its superior ability to mitigate the false positives (Type II errors) that are a key threat to validity in corporate disclosure analysis.

Finally, for the indicator construction, our validated model is deployed on the full corpus. To ensure maximum accuracy, we employ prompt engineering during the prediction phase. The model is specifically prompted to classify sentences based on concrete, realized corporate actions, and to ignore forward-looking or general statements. Based on its output, we construct our primary explanatory variable, *AI*, a dichotomous indicator that equals 1 if the model identifies at least one sentence with the “Applied” status in a given firm-year, and 0 otherwise. This construction ensures our indicator is a conservative and authentic measure of realized technological integration, not just corporate discourse. For robustness, we also construct a continuous variable, *AI_frequency*, measured as the frequency of these “Applied” sentences.

**Figure 1:**
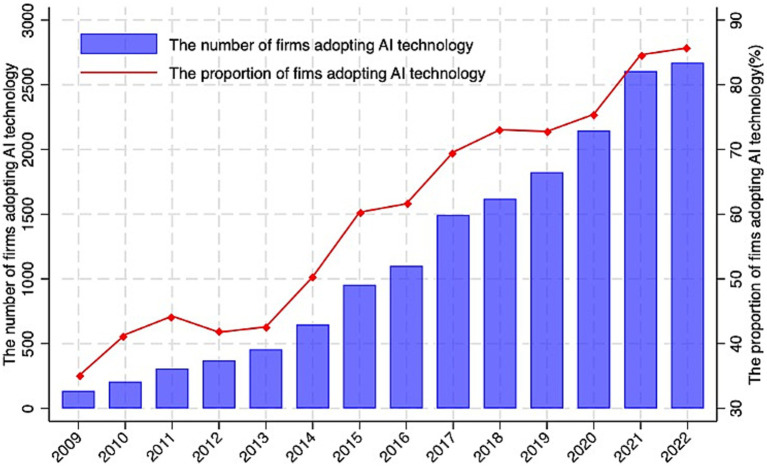
Trend of AI adopting among Chinese listed companies (2009–2022).

Figure 1 illustrates the overall AI adopting trend among Chinese listed companies from 2009 to 2022. The graph shows that the number of companies using AI technology is increasing year by year. Companies using AI technology have accounted for more than 80% of all Chinese listed companies. There have been more than 2,500 companies using AI technology in 2022, compared to no more than 500 a decade ago.

#### 3.3.3 Control variables

We include a standard set of control variables guided by the extensive literature on the determinants of corporate ESG performance (e.g., Gillan et al., 2021). These variables account for firm-specific characteristics that could confound the relationship between AI adoption and ESG outcomes. They include firm size (Size), firm age (Age), growth opportunities (Growth), profitability (ROA), leverage (Leverage), cash holdings (Cash), and several corporate governance characteristics: board size (Board), ownership concentration of the largest shareholder (Top1), and an indicator for CEO-chair duality (Dual). Detailed definitions and data sources for all variables are provided in Table 1.

**Table 1:** Variable definitions.

Variable	Definition	Data Source
ESG	Corporate ESG performance, measured by the Huazheng ESG rating index, converted to a numerical score from 1 (low) to 9 (high).	Wind
AI	A dichotomous indicator equal to 1 if a firm’s “Applied” AI technology is identified in a given year.	LLM-based text analysis of annual reports
AI_Frequency	A continuous measure of AI adoption, calculated as the number of sentences identified by our LLM as describing “Applied” AI technology, normalized by the total word count of the report.	LLM-based text analysis of annual reports
Size	The natural logarithm of the firm’s total assets.	CSMAR
Age	The number of years since the firm has been listed on the stock exchange.	CSMAR
Growth	The annual growth rate of the firm’s operating income.	CSMAR
ROA	Return on assets, calculated as net profit divided by total assets.	CSMAR
Cash	The ratio of cash and cash equivalents to total assets.	CSMAR
Board	The natural logarithm of the number of directors on the board.	CSMAR
Top1	The percentage of shares held by the firm’s largest shareholder.	CSMAR
Dual	An indicator variable equal to 1 if the CEO is also the board chair, and 0 otherwise.	CSMAR
Leverage	The ratio of total debt to total assets.	CSMAR
GDP	The GDP per capital of the city where the firm is located.	China Urban Statistical Yearbook
Population	The total population of the city where the firm is located.	China Urban Statistical Yearbook
College	The number of colleges and universities in the city where the firm is located.	China Urban Statistical Yearbook
Investment	The total amount of fixed assets invested in the city where the firm is located.	China Urban Statistical Yearbook

### 3.4 Summary statistics

The summary statistics for our sample of 22,931 firm-year observations are shown in Table 2. The ESG performance scores, our dependent variable, show a mean of 4.21 and range from 1 to 8, suggesting that the average firm in our sample has achieved an intermediate level of sustainability. However, the standard deviation of 1.026 indicates significant cross-firm variation in ESG practices. For our primary explanatory variable, AI adoption, the mean of 0.689 reveals a widespread, though not universal, adoption of AI technologies among Chinese listed firms. The substantial standard deviation (0.463) further highlights the heterogeneity in adoption intensity, with many firms still in the early stages of their technological integration.

**Table 2:** Descriptive statistics.

VarName	Obs.	Mean	SD	Min	Median	Max
ESG	22,931	4.210	1.026	1.000	4.250	8.000
AI	22,931	0.689	0.463	0.000	1.000	1.000
Size	22,931	22.226	1.300	19.894	22.032	26.298
Age	22,931	2.040	0.887	0.000	2.197	3.367
Growth	22,931	0.171	0.363	−0.500	0.114	2.114
Leverage	22,931	0.318	0.466	0.000	0.000	1.000
ROA	22,931	0.409	0.198	0.054	0.402	0.874
Cash	22,931	0.040	0.061	−0.233	0.040	0.206
Board	22,931	0.219	0.151	0.024	0.176	0.730
Top1	22,931	2.272	0.254	1.609	2.197	2.890
Dual	22,931	0.335	0.149	0.018	0.311	0.894

Further examination of the control variables reveals additional features. The average firm is large and established, with a mean log of total assets of 22.226. Corporate governance features include a notable concentration of ownership in the largest shareholder. Financially, the firms in our sample exhibit a median leverage of zero, suggesting that a significant portion of observations rely minimally on debt financing. The considerable variation in nearly all variables, as indicated by their standard deviations, underscores the diverse nature of the firms in our sample and confirms the suitability of our dataset for a large-sample fixed-effects analysis.

## 4 Results and discussion

This section presents our empirical findings. We begin by establishing the baseline relationship between AI adoption and ESG performance, then address potential endogeneity concerns with an instrumental variable approach. Subsequently, we confirm the robustness of our main finding through a battery of tests before examining the underlying mechanisms and heterogeneous effects.

### 4.1 Baseline regression

Table 3 presents the results of our baseline fixed-effects regressions. We progressively introduce control variables and fixed effects to ensure the stability of the relationship. Our preferred specification in Column (3), which includes a full set of controls and both firm and year fixed effects, shows a positive and statistically significant coefficient on our authentic AI adoption indicator (*β* = 0.033, *p* < 0.05). This provides initial support for Hypothesis H1, suggesting that the adoption of implemented AI technologies is associated with higher corporate ESG scores. To test for persistence, Column (4) uses a one-year lag of AI adoption; the coefficient remains positive and significant, indicating that the benefits of AI are not merely contemporaneous.

**Table 3:** Baseline regression results.

Variables	Dependent variable = *ESG*			
	(1)	(2)	(3)	(4)
AI	0.196***	0.135***	0.033**	
	(9.36)	(7.15)	(2.25)	
L.AI				0.032*
				(1.89)
Size		0.285***	0.347***	0.373***
		(22.83)	(15.50)	(13.70)
Age		−0.206***	−0.241***	−0.261***
		(−13.06)	(−9.24)	(−5.69)
Growth		−0.178***	−0.046***	−0.020
		(−8.83)	(−2.75)	(−1.03)
Leverage		−0.561***	−0.884***	−0.877***
		(−6.72)	(−10.54)	(−8.51)
ROA		2.528***	0.492***	0.261
		(12.99)	(3.19)	(1.55)
Cash		0.423***	0.165**	0.255***
		(5.36)	(2.23)	(2.98)
Board		−0.229***	−0.192***	−0.129***
		(−5.45)	(−6.50)	(−3.95)
Top1		0.147	0.498***	0.493***
		(1.60)	(3.35)	(2.77)
Dual		−0.062**	−0.021	−0.033
		(−2.54)	(−0.85)	(−1.21)
Constant	4.075***	−1.248***	−2.448***	−3.118***
	(208.19)	(−4.85)	(−5.02)	(−5.23)
Firm-fixed	Yes	No	Yes	Yes
Year-fixed	Yes	No	Yes	Yes
Observations	22,931	22,931	22,931	16,843
R-squared	0.008	0.152	0.657	0.706

This table presents the baseline fixed-effects regression results for the impact of AI adoption on ESG performance. The dependent variable is the firm’s ESG score. Column (1) includes only the AI indicator with fixed effects. Column (2) adds firm-level controls without fixed effects. Column (3) is our main specification, including all controls and both firm and year fixed effects. Column (4) tests the effect of the one-year lagged AI adoption variable (L.AI). All control variable definitions are in Table 1. *t*-statistics clustered by firms are in parentheses. ^***^, ^**^, and ^*^ denote significance at the 1, 5, and 10% levels, respectively.

The baseline regression results confirm that AI applications have enabled firms to cultivate a suite of distinctive organizational capabilities—most notably in ESG data analytics, risk early warning, and resource optimization. These AI-driven capabilities constitute valuable (V), rare (R), and inimitable (I) strategic resources, empowering firms to achieve superior ESG performance in key areas such as energy conservation and emissions reduction, supply chain transparency, and regulatory compliance. By embedding these capabilities into their operational and governance processes, firms secure a sustainable competitive advantage.

### 4.2 Addressing endogeneity

A key challenge in interpreting our baseline finding is the potential for endogeneity, particularly from reverse causality or omitted variables. For instance, firms with a stronger underlying commitment to sustainability might be more inclined to adopt forward-looking technologies like AI. To address this, we employ a two-stage least squares (2SLS) estimation using an instrumental variable (IV) strategy, with results presented in Table 4.

**Table 4:** Instrumental variable estimation results.

Variables	(1)	(2)
	AI	ESG
AI		1.260**
		(1.98)
AI_iv	0.187***	
	(3.54)	
Size	0.043***	0.294***
	(4.37)	(7.67)
Age	0.011	−0.251***
	(0.88)	(−8.22)
Growth	−0.004	−0.041**
	(−0.51)	(−2.03)
Leverage	−0.041	−0.830***
	(−1.02)	(−8.25)
ROA	0.174**	0.282
	(2.54)	(1.44)
Cash	−0.042	0.215**
	(−1.12)	(2.32)
Board	0.008	−0.201***
	(0.51)	(−5.77)
Top1	0.082	0.392**
	(1.26)	(2.28)
Dual	−0.000	−0.021
	(−0.00)	(−0.75)
Firm-fixed	Yes	Yes
Year-fixed	Yes	Yes
Kleibergen-Paap rk LM statistic		13.08***
Kleibergen-Paap Wald rk F statistic		12.56***
Observations	22,931	22,931

This table presents the 2SLS instrumental variable regression results. The dependent variable in the second stage is the firm’s ESG score. Column (1) reports the first-stage regression of a firm’s AI adoption on the instrumental variable (AI_iv), which is the leave-one-out industry-year mean of AI adoption. Column (2) reports the second-stage results for the effect of the instrumented AI adoption on ESG performance. All specifications include the full set of control variables and firm and year fixed effects. *t*-statistics clustered by firms are in parentheses. ^***^, ^**^, and ^*^ denote significance at the 1, 5, and 10% levels, respectively. The Kleibergen-Paap rk LM statistic tests for under identification, and the Kleibergen-Paap rk Wald F-statistic tests for a weak instrument.

Following a well-established approach in the corporate finance and economics literature, we instrument for a firm’s own AI adoption using the leave-one-out mean of AI adoption among all other firms in the same industry and year (Tan et al., 2022; Gormsen et al., 2024; Li et al., 2025). The validity of this instrument hinges on two conditions. First, for relevance, a firm’s technology choices are known to be strongly influenced by its peers due to competitive pressures, information spillovers, and shared industry trends (Leary and Roberts, 2014). The first-stage regression result in Column (1) of Table 4 confirms this strong positive association, with a Kleibergen-Paap F-statistic well above the conventional threshold for weak instruments. Second, for the exclusion restriction to hold, the industry-level adoption trend must not be correlated with the unobserved, firm-specific factors that drive an individual firm’s ESG performance, other than through its effect on that firm’s own adoption. After controlling for firm and year fixed effects which absorb time-invariant firm characteristics and common macroeconomic shocks, this condition is likely to be met.

The second-stage results in Column (2) show that the coefficient on the instrumented AI adoption variable remains positive and statistically significant. Notably, the magnitude of the 2SLS coefficient (1.260) is considerably larger than the OLS estimate. This is a common finding in IV estimations and can suggest that the OLS result is biased downward due to measurement error in the AI adoption proxy—an issue central to our paper’s motivation—or that the IV is identifying a Local Average Treatment Effect (LATE) for firms more responsive to industry trends. In either case, this 2SLS estimate confirms that our primary finding is robust to potential endogeneity from reverse causality.

### 4.3 Robustness tests

To further assess the robustness of our primary finding, we subject the baseline model to a series of additional tests, reported in Table 5. Each column represents a distinct check on the stability of our result.

**Table 5:** Additional robust tests.

Variables	(1)	(2)	(3)	(4)	(5)
	ESG	ESG	ESG	ESG_med	ESG
AI	0.034**		0.033***	0.033**	
	(2.27)		(2.95)	(2.11)	
L.AI		0.032*			
		(1.91)			
AI_frequency					0.810**
					(2.51)
Size	0.347***	0.374***	0.347***	0.356***	0.346***
	(15.48)	(13.67)	(26.46)	(15.45)	(15.54)
Age	−0.241***	−0.260***	−0.241***	−0.228***	−0.245***
	(−9.25)	(−5.69)	(−5.94)	(−8.47)	(−9.41)
Growth	−0.046***	−0.021	−0.046	−0.039**	−0.045***
	(−2.78)	(−1.05)	(−1.64)	(−2.20)	(−2.69)
Leverage	−0.880***	−0.875***	−0.884***	−0.889***	−0.883***
	(−10.52)	(−8.48)	(−8.27)	(−10.24)	(−10.57)
ROA	0.493***	0.265	0.492***	0.578***	0.508***
	(3.20)	(1.57)	(3.15)	(3.54)	(3.30)
Cash	0.164**	0.255***	0.165**	0.168**	0.170**
	(2.23)	(2.97)	(2.25)	(2.21)	(2.31)
Board	−0.192***	−0.129***	−0.192***	−0.198***	−0.191***
	(−6.51)	(−3.97)	(−6.91)	(−6.25)	(−6.45)
Top1	0.495***	0.493***	0.498***	0.564***	0.508***
	(3.34)	(2.77)	(3.52)	(3.64)	(3.41)
Dual	−0.021	−0.033	−0.021	−0.019	−0.021
	(−0.85)	(−1.21)	(−1.14)	(−0.76)	(−0.87)
Constant	−2.958**	−3.888***	−2.448***	−2.669***	−2.424***
	(−2.54)	(−2.80)	(−7.02)	(−5.33)	(−4.99)
City-control	Yes	Yes	No	No	No
Firm-fixed	Yes	Yes	Yes	Yes	Yes
Year-fixed	Yes	Yes	Yes	Yes	Yes
Observations	22,931	16,843	22,931	22,931	22,931
R-squared	0.657	0.706	0.657	0.627	0.657

This table reports the results of several robustness checks. The dependent variable in all columns is the firm’s ESG score, except in Column (4) where it is the yearly median ESG score. Column (1) adds city-level controls. Column (2) tests the effect of a one-year lagged AI adoption variable. Column (3) clusters standard errors at the industry level. Column (5) uses our continuous AI_frequency measure as the key explanatory variable. All specifications include the full set of control variables and firm and year fixed effects unless otherwise noted. *t*-statistics clustered by firms are in parentheses. ^***^, ^**^, and ^*^ denote significance at the 1, 5, and 10% levels, respectively.

First, in Column (1), we include additional city-level control variables. This addresses the concern that our results might be driven by unobserved regional economic or policy factors rather than firm-level AI adoption. The coefficient on AI remains positive and significant. Second, Column (2) confirms the persistent effect of AI using a lagged measure, mitigating concerns about strict simultaneity. Third, to address potential error correlation among firms subject to common industry-level shocks—a well-known issue in panel data analysis (Petersen, 2009)—we cluster our standard errors at the industry level in Column (3). This provides a more conservative estimate, and our main finding holds. Fourth, in Column (4), we use an alternative measure of the dependent variable, the yearly median ESG score, to ensure our finding is not an artifact of the specific construction of our primary ESG index. The result remains robust. Finally, in Column (5), we substitute our binary *AI* indicator with our continuous measure, *AI_frequency*. The positive and significant coefficient demonstrates that the effect is not just about whether a firm adopts AI, but that the intensity of authentic adoption also matters. The stability of our main finding across this battery of tests provides strong confidence that the positive AI-ESG relationship is a genuine empirical regularity.

### 4.4 Mechanism analysis

Having established a robust positive relationship, we now test the two causal channels proposed in Hypothesis H2 (Green Innovation) and Hypothesis H3 (Internal Control). Table 6 presents the results.

**Table 6:** Mechanism tests.

Variables	(1)	(2)	(3)	(4)
	*Green_pat*	*Green_pat*	*IC_index*	*IC_index*
AI	0.197***	0.017*	0.168***	0.081***
	(12.67)	(1.69)	(6.32)	(3.00)
Size	0.150***	0.070***	0.024	0.161***
	(7.86)	(4.02)	(1.40)	(3.77)
Age	−0.130***	0.014	0.828***	3.606***
	(−8.26)	(0.76)	(36.96)	(70.28)
Growth	−0.061***	−0.026***	0.087**	0.051
	(−3.83)	(−2.69)	(2.01)	(1.29)
Leverage	0.354***	−0.014	−0.785***	−1.506***
	(4.87)	(−0.26)	(−6.87)	(−8.07)
ROA	0.421***	0.088	2.050***	1.491***
	(2.80)	(1.09)	(7.19)	(4.50)
Cash	0.316***	−0.018	−1.134***	−0.236*
	(3.83)	(−0.36)	(−11.24)	(−1.69)
Board	−0.023	−0.029	−0.294***	−0.022
	(−0.49)	(−1.31)	(−6.27)	(−0.35)
Top1	−0.392***	−0.026	0.345***	3.139***
	(−4.11)	(−0.28)	(4.46)	(12.02)
Dual	0.042	0.008	0.113***	0.097**
	(1.64)	(0.52)	(4.66)	(2.40)
Constant	−2.822***	−1.074***	4.605***	−5.452***
	(−7.13)	(−2.83)	(14.51)	(−5.93)
Firm-fixed	No	Yes	No	Yes
Year-fixed	No	Yes	No	Yes
Observations	22,931	22,931	22,931	22,931
R-squared	0.070	0.717	0.172	0.531

This table presents the results for the mechanism tests outlined in Hypotheses H2a and H2b. Columns (1) and (2) test the green innovation channel, where the dependent variable is the number of green patent applications (Green_pat). Columns (3) and (4) test the internal control channel, where the dependent variable is the natural logarithm of the DIB Internal Control Index (IC_index). The main explanatory variable in all columns is our AI adoption indicator. Specifications (2) and (4) are our preferred models, which include firm fixed effects. All models include year fixed effects and the standard set of control variables. *t*-statistics clustered by firms are in parentheses. ^***^, ^**^, and ^*^ denote significance at the 1, 5, and 10% levels, respectively.

To test the green innovation channel, we use the number of green patent applications (*Green_pat*) as a proxy for a firm’s green innovation output. Column (2) shows that AI adoption has a positive and statistically significant effect on green patenting. This finding suggests that AI serves as a powerful enabling technology for corporate green R&D, likely by reducing the costs of experimentation and improving the efficiency of developing new sustainable processes and products. This evidence provides firm-level support for the broader proposition that technological advancement is a critical pathway to achieving sustainability goals, thus empirically supporting Hypothesis H2.

To test the internal control channel, we use the natural logarithm of the DIB Internal Control Index (*IC_index*), following prior research in the Chinese context (Yang et al., 2024). The results in Column (4) show that the coefficient on AI is consistently positive and significant, providing strong support for Hypothesis H3. This result highlights AI’s role as a governance-enhancing technology. From a corporate governance perspective, this suggests that AI can serve as a powerful tool to mitigate agency problems. By creating a more transparent and controlled internal environment through real-time data analysis and anomaly detection, AI enhances the board’s oversight capabilities, reduces information asymmetry, and helps ensure that managerial actions align with the long-term sustainability interests of shareholders and other stakeholders.

From an RBV perspective, an AI-driven, highly intelligent internal control system constitutes a distinctive organizational capability that is inherently difficult to imitate. Likewise, AI-enabled, data-driven R&D processes represent valuable resources that strengthen firms’ capacity for green innovation. Accordingly, firms that effectively harness AI and embed it deeply within their internal control and green innovation processes can enhance ESG performance in ways that are hard for competitors to replicate, thereby achieving a sustainable competitive advantage.

In summary, our mechanism analysis provides robust evidence that AI adoption facilitates superior corporate ESG performance not in a vacuum, but through the tangible pathways of fostering green innovation and enhancing the quality of internal control.

### 4.5 Heterogeneity analysis

The diffusion and impact of new technologies are rarely uniform across firms, a well-documented empirical regularity in the economics of innovation (Comin and Hobijn, 2010). Our theoretical framework, grounded in RBV and TOE, leads to Hypothesis H2, which predicts that the positive effect of AI on ESG will be more pronounced in firms possessing greater resources and higher absorptive capacity. We test this by partitioning our sample along two key dimensions: industry technological intensity and firm size. The results of these tests are reported in Table 7.

**Table 7:** Heterogeneity analysis.

Variables	Dependent variable = *ESG*				
	(1)	(2)	(3)	(4)	(5)
	Labor intensity	Capital intensity	Technology intensity	Large firms	Small firms
AI	0.013	0.030	0.043**	0.065***	0.011
	(0.52)	(0.83)	(2.03)	(3.28)	(0.48)
Size	0.346***	0.454***	0.343***	0.414***	0.405***
	(8.33)	(6.36)	(10.54)	(11.11)	(10.17)
Age	−0.166***	−0.403***	−0.254***	−0.043	−0.283***
	(−3.41)	(−5.14)	(−7.80)	(−0.87)	(−7.85)
Growth	−0.019	−0.076*	−0.069***	−0.049**	−0.003
	(−0.63)	(−1.67)	(−3.00)	(−2.03)	(−0.12)
Leverage	−0.768***	−1.266***	−0.751***	−0.963***	−0.915***
	(−4.97)	(−5.82)	(−6.53)	(−6.67)	(−8.49)
ROA	0.608**	−0.367	0.562***	0.657***	−0.038
	(2.23)	(−0.88)	(2.75)	(2.64)	(−0.20)
Cash	0.118	−0.514**	0.242**	0.224*	0.096
	(0.94)	(−2.22)	(2.51)	(1.85)	(1.04)
Board	−0.149***	−0.225***	−0.189***	−0.146***	−0.171***
	(−3.12)	(−2.68)	(−4.68)	(−3.50)	(−4.23)
Top1	0.269	−0.047	0.430*	0.127	1.193***
	(1.16)	(−0.14)	(1.83)	(0.61)	(4.93)
Dual	−0.064	−0.041	−0.003	−0.012	−0.037
	(−1.47)	(−0.53)	(−0.08)	(−0.35)	(−1.10)
Constant	−2.630***	−3.980**	−2.373***	−4.516***	−3.746***
	(−2.93)	(−2.48)	(−3.29)	(−5.30)	(−4.51)
Observations	7,110	3,025	12,485	11,319	11,292
R-squared	0.706	0.665	0.651	0.685	0.676

This table reports the results of the heterogeneity analysis. The dependent variable is the firm’s ESG score. Columns (1)–(3) present results for subsamples based on industry factor intensity. Columns (4) and (5) present results for subsamples based on a median split of firm size. All specifications include the full set of control variables and firm and year fixed effects. *t*-statistics clustered by firms are in parentheses. ^***^, ^**^, and ^*^ denote significance at the 1, 5, and 10% levels, respectively.

First, we examine the moderating role of industry factor intensity. Following prior research on innovation in the Chinese context (Lu and Dang, 2014), we classify firms into technology-intensive, capital-intensive, and labor-intensive categories based on the 2012 industry classification standards of the China Securities Regulatory Commission. The results are striking: the positive and statistically significant effect of AI on ESG is exclusively concentrated in technology-intensive firms (Column 3), while the coefficients for both labor-intensive (Column 1) and capital-intensive (Column 2) firms are statistically insignificant.

The positive effects of AI adoption are particularly pronounced in technology-intensive enterprises. The fundamental reason is that these firms already possess the complementary assets and absorptive capacity required to effectively assimilate and deploy AI as an emerging strategic resource.

From an RBV perspective, technology-intensive enterprises typically command high-quality teams of engineers and data scientists, advanced R&D facilities, and robust data infrastructures. These resources are highly complementary to AI, which does not generate value in isolation but must be integrated with a firm’s existing technological base. Moreover, due to their long-standing technological accumulation and R&D experience, such firms possess stronger absorptive capacity. They are better equipped to interpret and evaluate AI’s evolving knowledge system and to embed it effectively into internal controls and product innovation processes. Meanwhile, this finding also lends strong empirical support to the TOE framework. It suggests that a firm’s pre-existing technological and organizational context is a critical precondition for success. Technology-intensive firms, by their nature, possess a deep reservoir of technical expertise and established R&D routines, which constitutes a high level of “absorptive capacity” (Cohen and Levinthal, 1990). This capacity allows them to more effectively understand, integrate, and exploit AI’s potential for complex operational processes related to environmental monitoring and corporate governance.

Second, we test the moderating role of firm size, a proxy for a firm’s resource endowment. The evidence indicates that the sustainability benefits of AI are similarly concentrated among large firms (Column 4), while the effect for smaller firms is statistically insignificant (Column 5). This result is highly consistent with the RBV (Barney, 1991). The value of a new technology is often unlocked only when it is combined with other supportive firm resources, or “complementary assets” (Teece, 1986). In this context, large firms possess these crucial assets in greater abundance—including vast, proprietary datasets to train models, the financial capital to absorb the long-term and often uncertain returns of ESG-related projects, and the specialized human capital required to manage both the technology and the complex ESG reporting landscape. The absence of these critical complementary assets appears to be a binding constraint, limiting the ability of smaller firms to operationalize AI for tangible ESG gains. Furthermore, from a TOE perspective, large-scale enterprises typically possess more mature organizational structures and managerial processes that facilitate the adoption of new technologies. Such firms are often able to establish dedicated AI departments, implement systematic employee training programs, and develop standardized application procedures. These well-developed organizational mechanisms not only reduce internal resistance to AI implementation but also accelerate its diffusion across business units, thereby amplifying AI’s positive impact on ESG performance.

## 5 Conclusion

This study investigates the critical relationship between artificial intelligence and corporate ESG performance, addressing a core challenge of authentic measurement. Our approach advances the foundational LLM-based framework of Jin et al. (2024); by deploying a highly capable model well-suited for Chinese text (Qwen2.5-72B) and, more importantly, by introducing a granular classification scheme designed to isolate substantive technological applications from superficial corporate discourse. Leveraging this improved measure, our empirical analysis of Chinese A-share firms from 2009 to 2022 yields several robust findings. We document a significant positive linkage between authentic AI adoption and corporate ESG performance, an effect that is channeled through two primary pathways: the fostering of corporate green innovation and the strengthening of internal control quality. Furthermore, our heterogeneity analysis reveals that this positive effect is concentrated in larger and more technology-intensive firms. This finding provides strong empirical support for our theoretical framework, aligning with both the RBV and TOE perspectives.

These findings yield important practical and policy implications, particularly in the context of escalating institutional pressures. For corporate leaders, our results show that AI is a strategic asset for achieving sustainability. However, our analysis of adoption challenges underscores that the path to value creation lies in responsible implementation. Managers should champion AI integration with a clear vision for its role in improving environmental monitoring and supply chain ethics, while simultaneously building a robust AI governance structure aligned with principles of “Trustworthy AI” to manage its inherent risks.

For policymakers, our findings offer timely insights that can inform the next phase of China’s evolving sustainability disclosure regime. The introduction of mandatory reporting in 2024 by China’s three major exchanges marks a critical milestone, moving decisively beyond the voluntary guidance of 2006–2008. However, our results on the challenges of AI adoption and the emerging “digital ESG divide” highlight where future policy must focus. To ensure the success of the new mandatory framework and to prevent it from disproportionately burdening smaller firms, policies should be designed to lower the barriers to AI entry. This could include targeted subsidies to mitigate the high implementation costs, promoting the adoption of official AI risk management frameworks (National Institute of Standards and Technology (US) and U.S. Department of Commerce, 2024) to address governance risks, and public investment in upskilling the workforce to address the critical talent bottleneck. In an era where new regulations like the EU’s AI Act are setting global standards for technology governance, such proactive policies are crucial for aligning national digital transformation with global sustainability and governance norms (European Union, 2024).

While this study provides valuable insights, its limitations open several promising avenues for future research. Our analysis is situated in a single country; future cross-country studies are needed to test the external validity of our findings. More pointedly, future research could leverage emerging AI techniques to deepen our understanding. First, the application of Explainable AI (XAI) could open the “black box” of more complex models to better understand the specific ESG factors that firm-level AI systems are optimizing (Bussmann et al., 2025). Second, advanced NLP models could be designed to detect and quantify corporate “greenwashing” in sustainability disclosures (Freunek and Niggli, 2023). Finally, the use of synthetic data could help address data scarcity issues, enabling more robust testing of AI’s impact in data-poor sustainability contexts (Tkachenko, 2024).

## Data Availability

The original contributions presented in the study are included in the article/supplementary material, further inquiries can be directed to the corresponding author.

## Footnotes

^1^Qwen2.5-72B is a large language model from the Tongyi Qianwen (Qwen) family, developed by Alibaba Cloud and released in February 2024. The model features 72 billion parameters and is recognized for its advanced capabilities in both Chinese and English language understanding and generation, making it particularly well-suited for analyzing our Chinese-language corporate disclosures.
